# Chronic suppression of monoacylglycerol lipase restores adult neurogenesis in the septal but not the temporal DG in Ts65Dn mouse model of Down syndrome

**DOI:** 10.1016/j.brainresbull.2026.111721

**Published:** 2026-01-08

**Authors:** Donya Fozoonmayeh, Mathangi Sankaran, Jessica Yu, Meriel Walsh, Anna Tyrtyshnaia, Alexander M. Kleschevnikov

**Affiliations:** Department of Neurosciences, University of California San Diego, 9500 Gilman Drive, La Jolla, CA 92093, USA

**Keywords:** Genetic models, Endocannabinoid system, 2-Arachidonoil glycerol, Monoacylglycerol lipase, JZL184, Adult neurogenesis, Dentate gyrus, Septal, Temporal

## Abstract

Down syndrome (DS) is a genetic disorder characterized by cognitive impairment and varying degrees of changes in emotion-related behaviors. A deficiency in adult hippocampal neurogenesis is among the cellular mechanisms implicated in both abnormalities. Previously, we observed that chronic inhibition of monoacylglycerol lipase (MAGL) with the selective inhibitor JZL184 increased brain levels of the endocannabinoid 2-arachidonoylglycerol (2-AG), improved hippocampal synaptic plasticity and long-term memory, but did not affect anxiety-related thigmotactic behavior in Ts65Dn mice, a genetic model of DS. In this study, we tested the hypothesis that these effects of JZL184 might be associated with changes in adult hippocampal neurogenesis. Ts65Dn mice and their normosomic (2 N) littermates were injected daily for 3 weeks with JZL184 or vehicle, and bromodeoxyuridine (BrdU) was co-administered during the chronic phase of the treatment. BrdU-immunopositive cells were quantified in the septal, medial, and temporal segments of the dentate gyrus (DG). It was observed that both the total number and the density of BrdU-positive cells were significantly reduced in Ts65Dn mice compared to their 2 N littermate controls. Strikingly, JZL184 treatment effects exhibited a profound septo-temporal bias: the BrdU-immunopositive cell density was restored to near control levels in the septal DG (a region presumably linked to cognitive function), but it was largely unaffected in the temporal DG (presumably associated with emotion-related behaviors). These results suggest that chronic MAGL inhibition may provide a targeted region-specific therapeutic strategy for cognitive impairment in Down syndrome, potentially independent of its effects on emotional behavior.

## 1. Introduction

Down syndrome (DS), also known as trisomy 21 (Lejeune et al., 1959), results in moderate to severe cognitive impairment and a variety of changes in emotion-based behaviors (Antonarakis et al., 2020; Belichenko et al., 2015; Hamadelseed et al., 2023; Rafii et al., 2019; Robinson et al., 2024; Shaffer et al., 2023). These abnormalities arise primarily from late prenatal and early postnatal neurodevelopmental alterations that profoundly affect cell number, circuit formation, and neuronal connectivity in the hippocampus and other brain regions (Guidi et al., 2008; Ishihara, 2021; Russo et al., 2024; Stagni et al., 2018), while impairments in adult hippocampal neurogenesis are thought to further exacerbate hippocampus-dependent cognitive dysfunction (Belichenko and Kleschevnikov, 2011; Bianchi et al., 2014; Clark et al., 2006; Contestabile et al., 2013; Corrales et al., 2014; Latchney et al., 2015; Llorens-Martin et al., 2010; Lopez-Hidalgo et al., 2016; Martinez-Cue et al., 2013; Stagni et al., 2015; Trazzi et al., 2014; Velazquez et al., 2013).

Mouse genetic models have been successfully used to explore the cellular mechanisms of behavioral abnormalities in DS (Chang et al., 2025; Das and Reeves, 2011; Dierssen, 2012; Edgin et al., 2012; Herault et al., 2012; Kleschevnikov, A.M., 2022; Kleschevnikov et al., 2012), and to evaluate prospective treatments to improve impaired brain functions (Alasmari et al., 2025; Bartesaghi et al., 2022; Chen et al., 2024; Contestabile et al., 2013; Corrales et al., 2014; Latchney et al., 2015; Martinez Cue and Dierssen, 2020; Powers et al., 2017). The most extensively examined model of DS, Ts65Dn mice (Davisson et al., 1993), exhibit impaired synaptic plasticity and cognition (Belichenko et al., 2009; Costa and Grybko, 2005; Kleschevnikov et al., 2012, 2004; Siarey et al., 1997, 2005), altered emotion-related behaviors (Escorihuela et al., 1998; Martinez-Cue et al., 2005, 2006; Moon et al., 2010), and other changes similar to those observed of DS (Chakrabarti et al., 2007; Giacomini et al., 2015; Jin et al., 2025). Importantly, adult hippocampal neurogenesis is impaired in Ts65Dn mice and is thought to contribute, together with other neuronal and synaptic abnormalities, to their behavioral deficits (Belichenko and Kleschevnikov, 2011; Bianchi et al., 2014; Clark et al., 2006; Contestabile et al., 2013; Corrales et al., 2014; Latchney et al., 2015; Llorens-Martin et al., 2010; Lopez-Hidalgo et al., 2016; Martinez-Cue et al., 2013; Stagni et al., 2015; Strupp et al., 2016; Trazzi et al., 2014; Velazquez et al., 2013). Consequently, restoring adult neurogenesis through various therapeutic interventions has been shown to improve impaired brain functions and correct some of the behavioral abnormalities in these models (Contestabile et al., 2013; Islam et al., 2025; Latchney et al., 2015; Martinez-Cue et al., 2013; Moreau et al., 2024; Parrini et al., 2017; Strupp et al., 2016; Velazquez et al., 2013).

Given the critical role of adult neurogenesis in hippocampal function (Aimone et al., 2014; Baptista and Andrade, 2018), finding new strategies to restore it is an extremely important task. One such promising strategy is the modulation of the endocannabinoid system, known for its widespread influence on the development and plasticity of the nervous system (de Oliveira et al., 2019; Marino et al., 2025; Oddi et al., 2023; Oudin et al., 2011; Rivera et al., 2015; Zhang et al., 2015). Tissue levels of endocannabinoids are tightly controlled by enzymes responsible for their synthesis and degradation. For instance, inhibiting monoacylglycerol lipase (MAGL), the primary enzyme responsible for hydrolyzing the endocannabinoid 2-arachidonoylglycerol (2-AG), leads to a several-fold increase in brain 2-AG levels in mice (Kinsey et al., 2009). In our previous study, we demonstrated that treatment with JZL184, a selective MAGL inhibitor, significantly (3–4 fold) elevates brain 2-AG levels while also enhancing synaptic plasticity and cognitive function in adult Ts65Dn mice (Lysenko et al., 2014). These beneficial effects may, in part, be mediated by an increase in adult hippocampal neurogenesis. In the present study, we examined the impact of chronic MAGL inhibition on adult hippocampal neurogenesis in Ts65Dn mice. Our findings support the hypothesis that improved adult hippocampal neurogenesis may contribute to the enhancement of cognitive function observed in JZL184-treated Ts65Dn mice, as suggested by our previous work.

## 2. Materials and methods

### Animals.

Segmental trisomy 16 (Ts65Dn) mice were generated by mating female carriers of the 17^16^ chromosome (B6EiC3H-a/A-Ts65Dn) with (C57BL/6JEi X C3H/HeJ) F1 males (JAX #JR1875) as described (Davisson et al., 1993). Euploid (2 N) littermate mice served as controls. Mice were housed in groups of 2–4 per cage under a 12-hour light-dark cycle with *ad libitum* access to food and water. For genotyping, genomic DNA was extracted from tail samples according to protocol (Truett et al., 2000), and PCR analysis was performed according to protocol (Duchon et al., 2011). All mice were also screened for retinal degeneration due to *Pde6brd1* homozygosity (Bowes et al., 1993). The experiments were conducted in accordance with the National Institutes of Health guidelines and with an approved protocol from the University of California San Diego (UCSD) Institutional Animal Care and Use Committees.

### Experimental Design.

Littermate 9-month-old Ts65Dn and 2 N male mice were used in the study. Animals of each genotype were randomly assigned to one of two groups: one group received an intraperitoneal (i.p.) injection of JZL184 (8 mg/kg), and the other group received an equivalent volume of vehicle (saline, 10 mL/kg body mass). Injections were administered daily for 20 consecutive days. To label proliferating cells, 5-bromo-2′-deoxyuridine (BrdU, 10 mg/mL in saline; Sigma-Aldrich Corp., St. Louis, MO, USA, Cat. #B9285) was administered once daily as a single i.p. injection at a dose of 100 mg/kg body mass on days 12, 13, and 14 of the JZL184 or vehicle treatment. This three-day labeling window was chosen to mark actively proliferating neural stem cells and type 2/3 progenitors during the chronic phase of drug exposure, and the 7-day interval between the last BrdU injection and perfusion allowed assessment of both proliferation and early survival/commitment of newly generated cells. The mice were sacrificed on day 21, 24 h after the last injection of either JZL184 or vehicle. Thus, four groups of animals were examined: 2 N Vehicle (2 N Veh, n = 5), 2 N JZL184 (2 N JZL, n = 5), Ts65Dn Vehicle (Ts Veh, n = 5), and Ts65Dn JZL184 (Ts JZL, n = 4).

### Immunohistochemistry.

Mice were deeply anesthetized with ketamine/xylazine (10:1) mixture (150 mg/kg, i.p.) and transcardially perfused with 10 mL of 0.9 % NaCl for 1 min, followed by 10 min of perfusion with 4 % paraformaldehyde in 0.1 M phosphate-buffered saline (PFA-PBS), pH 7.4. The brains were carefully removed, post-fixed in 4 % PFA-PBS for 4–5 days at 4°C, then embedded in optimal cutting temperature (OCT) medium (Fisher Scientific, Cat. #23–730–571), and cut into 40 μm-thick coronal brain sections using a cryostat microtome (Leica CM3050S, Leica Biosystems). Every sixth section was selected for immunohistochemistry. For antigen (BrdU) retrieval, free-floating sections were incubated in 2 N hydrochloric acid for 30 min at 37°C, followed by neutralization in borate buffer (pH 8.4) for 10 min. Sections were blocked for 2 h in 5 % normal horse serum (NHS) and incubated overnight with sheep polyclonal anti-BrdU primary antibody (Abcam; Cat. #ab1993) at a 1:250 dilution in PBS. After 3–5 washes in PBST, sections were incubated the next morning with donkey anti-sheep Alexa Fluor 488 secondary antibody (Abcam, Cat. #ab150177). Subsequently, sections were counterstained with DAPI (Sigma Aldrich, Cat. #D9542) and washed 3–5 times in PBS. Finally, sections were mounted and cover-slipped using Fluoromount-G (ThermoFisher Scientific, Cat. #00–4958–02). All hippocampal sections from both 2 N and Ts65Dn mice were processed simultaneously by an operator blinded to genotype and treatment.

### Microscopy.

Brain sections were imaged using a CCD camera (Rolera-MGi Fast 1397, Qimaging) mounted on a fluorescent microscope (Leica DMI6000B, Leica Biosystems), operated with Leica Application Suite (Leica, Germany) and MetaMorph imaging software (Molecular Devices, Sunnyvale, CA). Each examined DG section was scanned through its entire volume using a Z-series stack with a 2 μm step size, chosen to ensure optimal resolution while minimizing photobleaching. The resulting images were flattened. Images from different DG regions were merged into a complete DG image using Photoshop (Adobe Systems, San Jose, CA, USA). Image analysis was subsequently performed using ImageJ (NIH, USA).

### Gross-morphological parameters of DG.

The volume and width of the granule cell layer (GCL) were measured to evaluate potential structural changes in the DG associated with genotype and/or treatment. The GCL volume was determined by measuring the GCL area in each coronal DG section. These area measurements were summed across all analyzed sections for each mouse, and the results were expressed as a percentage relative to the mean value of the 2 N Veh group. The suprapyramidal and infrapyramidal blades of the dentate gyrus were delineated in each coronal section based on their relationship to the hippocampal fissure and the CA1/subiculum border: in rostral and intermediate sections, the suprapyramidal blade was defined as the portion of the GCL located dorsal to the hippocampal fissure and adjacent to CA1, whereas the infrapyramidal blade was defined as the portion ventral to the fissure and oriented toward the hilus/CA3; in more caudal sections, where curvature complicates this distinction, the apex of the GCL and the trajectory of the fissure were used as landmarks, with the suprapyramidal blade extending from the apex toward dorsal CA1 and the infrapyramidal blade from the apex toward the ventral hilus/CA3. To calculate the average GCL width, measurements were taken at three predefined locations (apex, middle, and base) along both the suprapyramidal and infrapyramidal blades of the DG for each section. These measurements were averaged to obtain a mean GCL width for each mouse, and group means were calculated by averaging the values across all animals in each experimental group. Image analysis, including area and width measurements, was conducted using ImageJ software (National Institutes of Health, USA). All analyses were performed by an experimenter blinded to the genotype and treatment.

### Cell quantification.

BrdU-positive cells were quantified by counting all BrdU-immunopositive cells in the subgranular zone (SGZ) of each DG section, with the total count obtained by summing the counts from each section. Since every sixth section was analyzed, the final count was multiplied by 6. To evaluate the distribution of BrdU-immunopositive cells across DG subregions, linear density was calculated by counting cells within 25 μm of the hilus/GCL border and dividing by the length of the border. This approach ensures an accurate description of cell distribution in a heterogeneous structure like the DG, as most of newly born cells in adult mice are located near the hilus/GCL border (Ansorg et al., 2012). These values were averaged across all sections for the entire DG or specific DG segments. To examine septo-temporal differences, the sections were grouped into three regions: rostral (predominantly septal, sections 64–69 based on the Allen Reference Atlas for the mouse brain, http://atlas.brain-map.org/), intermediate (sections 70–81), and caudal (predominantly temporal, sections 82–90). This tripartite classification was chosen to provide a more nuanced analysis of potential regional differences along the septo-temporal axis, as it approximates DG parcellation based on gene expression and brain-wide connectivity (Bienkowski et al., 2018). For simplicity, these DG segments are referred to as septal, intermediate, and temporal. All measurements and analyses were performed by an experimenter blinded to the genotype and treatment conditions.

### Statistics.

All datasets were examined for normality of distributions using the Shapiro-Wilk test and passed this test. The data were then evaluated for outliers using Dixon’s Q test, and no outliers were identified. Non-parametric Kruskal-Wallis test was used to assess differences between groups. When significant differences were detected, post-hoc pairwise comparisons were performed using the Mann-Whitney U-test. The differences were considered significant at p < 0.05. The data are presented as mean ± SEM.

## 3. Results

### 3.1. Effect of treatment on body weight and brain weight

Given that the endocannabinoid 2-AG is known to influence appetite and energy metabolism, we first assessed whether chronic JZL184 treatment affected body weight in our experimental groups. This analysis was important to rule out potential confounding effects of altered body mass on subsequent neurogenesis measurements. We compared post-treatment body weights across genotypes and treatment conditions. Body weight was lower in Ts65Dn mice compared to 2 N control littermates both in vehicle-treated (2 N Veh: 43.63 ± 1.87 g; Ts Veh: 37.41 ± 0.69 g, p < 0.03) and JZL184-treated (2 N JZL: 40.71 ± 1.26 g; Ts JZL: 35.82 ± 2.32 g, p < 0.03) groups. There was no significant difference in body weight between the vehicle- and JZL184-treated groups within either the 2 N (p = 0.18) or Ts65Dn (p = 0.48) groups of mice. Pooled together, the body weight of 2 N mice was approximately 13 % greater than that of Ts65Dn littermates, consistent with previous findings (2 N: 42.17 ± 1.3 g; Ts65Dn: 36.61 ± 1.09 g; p < 0.003). Total brain weight, in contrast, was not significantly affected by genotypes or treatment in our experimental groups (2 N Veh: 395.0 ± 24.3 mg; 2 N JZL: 407.5 ± 35.4 mg; Ts Veh: 425.0 ± 21.3 mg, Ts JZL: 427.5 ± 20.7 mg; p = 0.28–0.93 for all between-group comparisons). Thus, Ts65Dn mice exhibited lower body weights compared to 2 N controls, and JZL184 treatment did not significantly affect body weight in either genotype.

### 3.2. Changes in dentate gyrus neurogenesis and GCL morphology

**Fig. 1.**
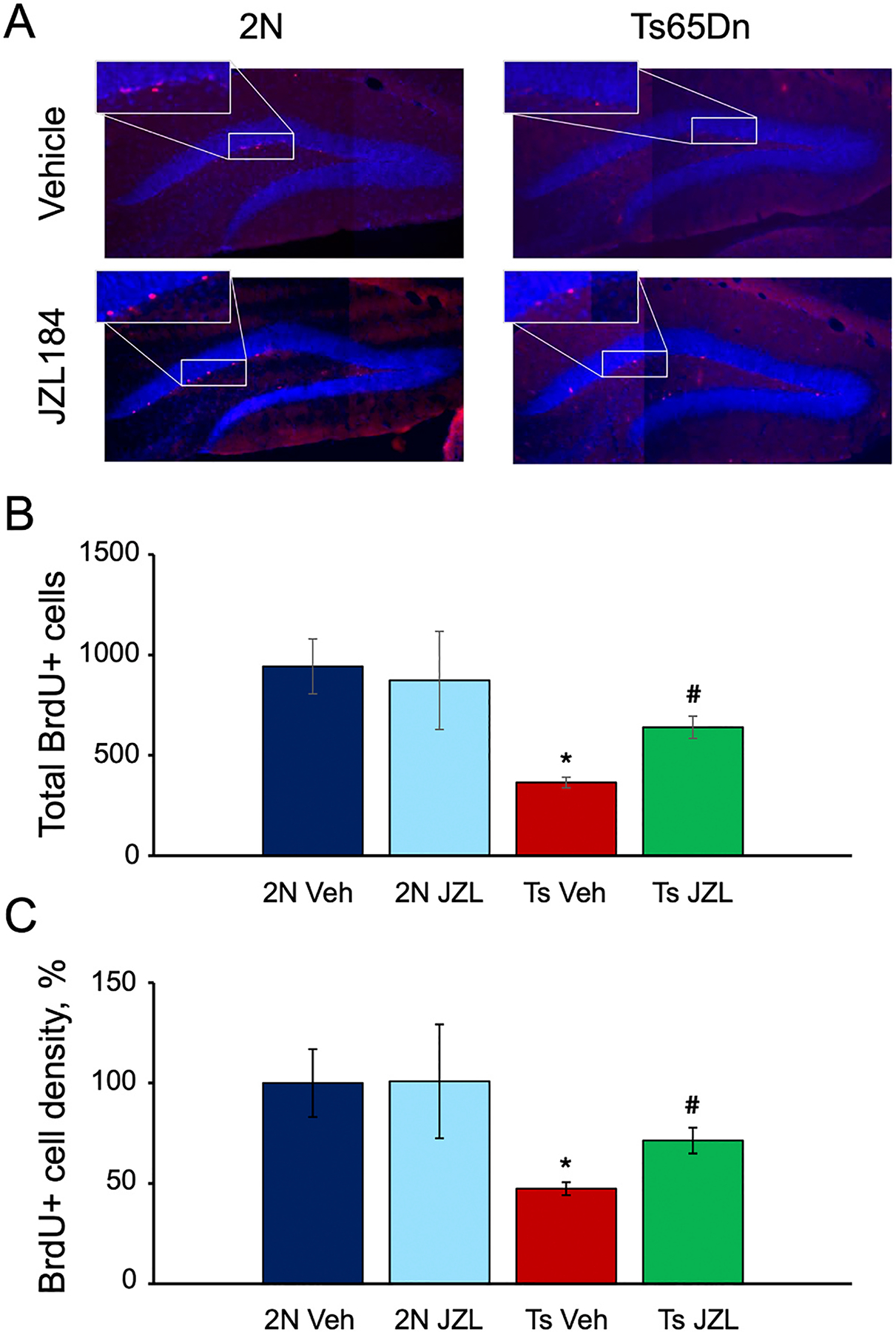
Chronic treatment with JZL184 improved adult neurogenesis in Ts65Dn DG. A. Representative images of DG sections co-immunostained for BrdU (red) and DAPI (blue). The number of BrdU-positive cells was reduced in the DG of vehicle-treated Ts65Dn *vs*. 2 N mice and partially restored in the DG of JZL184-treated Ts65Dn mice. **B, C.** Quantification of the data. Both the total number (**B**) and linear density (**C**) of BrdU-positive cells was significantly reduced in Ts65Dn Veh *vs*. 2 N Veh DG (red *vs*. dark blue) and increased in Ts65Dn JZL *vs*. Ts65Dn Veh DG (green *vs*. red). * p < 0.05, compared with 2 N Veh; # p < 0.05, compared with Ts Veh.

Deficient adult neurogenesis is a well-established characteristic of mouse models of DS. To assess the effects of JZL184 treatment, we quantified the total number of BrdU-immunopositive cells in the DG of each animal. BrdU-positive cells were clearly visible along the border of the granule cell layer and hilus in DG sections co-immunostained for BrdU and DAPI (Fig. 1A). Quantification revealed a significantly reduced total number of BrdU-positive cells in the vehicle-treated Ts65Dn DG compared to 2 N DG (Fig. 1B, red *vs*. dark blue). JZL184 treatment increased the total number of BrdU-positive cells in the Ts65Dn DG (Fig. 1B, green *vs*. red) but not in 2 N control mice (Fig. 1B, light blue *vs*. dark blue).

We next assessed the density of BrdU-positive cells along the granule cell layer/hilus border. Similarly to the total BrdU cell number, density was significantly reduced in vehicle-treated Ts65Dn mice compared to 2 N mice (Fig. 1C, red *vs*. dark blue). The JZL184 treatment increased BrdU cell density in Ts65Dn mice (Fig. 1C, green *vs*. red) but not in 2 N mice (Fig. 1C, light blue *vs*. dark blue). Thus, adult neurogenesis was significantly impaired in the DG of Ts65Dn mice, and this impairment was partially reversed by JZL184 treatment.

Changes in adult neurogenesis could potentially affect gross-morphological parameters of the DG granule cell layer (GCL). To assess this, GCL volume and width were compared across groups (Table 1). GCL volume was significantly reduced in vehicle-treated Ts65Dn mice but was restored to control levels by JZL184 treatment. This volume increase was selective for Ts65Dn mice, with no significant effect in 2 N mice. GCL width was similar across all groups and unaffected by genotype or treatment. Thus, reduced adult neurogenesis in Ts65Dn mice was accompanied by reduced GCL volume, while GCL width was unaffected; JZL184 treatment normalized GCL volume in Ts65Dn mice.

### 3.3. Regional differences along the septo-temporal axis

**Fig. 2.**
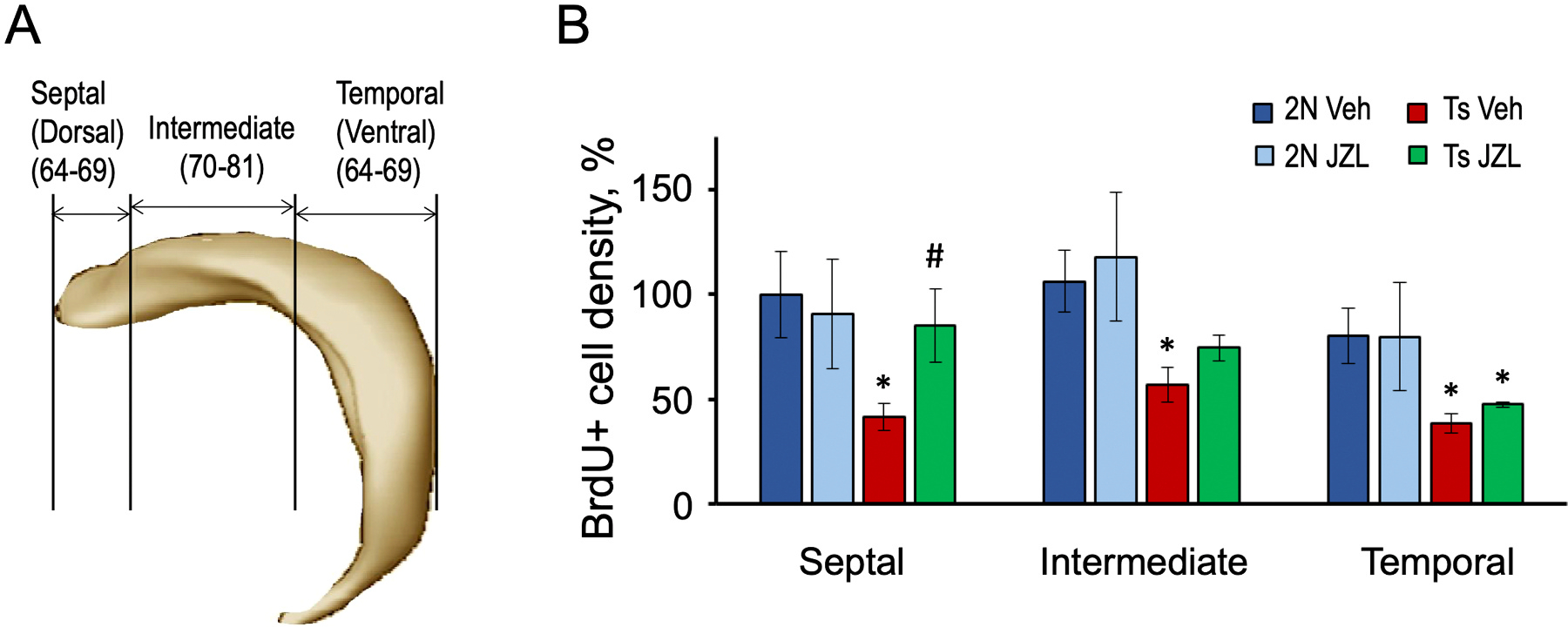
Septo-temporal differences in the effects of JZL184 treatment on adult neurogenesis. A. A schematic showing the division of DG into rostro-septal, intermediate, and caudo-temporal segments. The numbers in parentheses correspond to the rostro-caudal position of sections in the Allen mouse brain atlas. **B.** Densities of BrdU-positive cells in the corresponding DG segments. The BrdU cell density was evenly reduced in all DG segments of Ts Veh *vs*. 2 N Veh mice. JZL184 treatment fully restored the BrdU cell density in the rostro-septal segment but had no effect in the caudo-temporal DG segment of Ts65Dn mice. In 2 N mice, JZL184 treatment had no effect on the BrdU cell density in all three DG segments. * p < 0.05, compared with 2 N Veh; # p < 0.05, compared with Ts Veh.

The septal (dorsal) and temporal (ventral) parts of the hippocampus differ in their involvement in the higher brain functions and in multiple molecular, synaptic, and neuronal properties. To assess the septo-temporal differences in adult neurogenesis and the effects of JZL184 treatment, the dentate gyrus of each animal was divided into septal, intermediate, and temporal segments (Fig. 2A). In all three segments, BrdU-positive cell density was significantly lower in Ts Veh compared to 2 N Veh (Fig. 2B, red *vs*. dark blue), reaching 41.6 ± 6.3 %, 53.6 ± 7.7 %, and 47.8 ± 5.8 % of the corresponding 2 N Veh values in the septal, intermediate, and temporal DG, respectively. Thus, adult neurogenesis was markedly reduced across all septo-temporal segments in Ts65Dn mice.

In Ts65Dn mice, JZL184 treatment significantly increased BrdU-positive cell density in the septal DG to levels not significantly different from 2 N Veh (Fig. 2B, green). In the intermediate DG, JZL184 increased cell density numerically, but the change did not reach statistical significance. In the temporal DG, treatment had no effect, with density remaining at Ts Veh levels and significantly lower than in 2 N mice. Expressed as percentages of the corresponding 2 N Veh values, BrdU-positive cell densities in Ts JZL mice were 85.4 ± 17.6 %, 70.2 ± 5.7 %, and 59.5 ± 1.6 % in the septal, intermediate, and temporal segments, respectively. Thus, the effect of JZL184 on adult neurogenesis in Ts65Dn mice was mainly restricted to the septal DG segment.

### 3.4. Suprapyramidal vs. Infrapyramidal DG blades

**Fig. 3.**
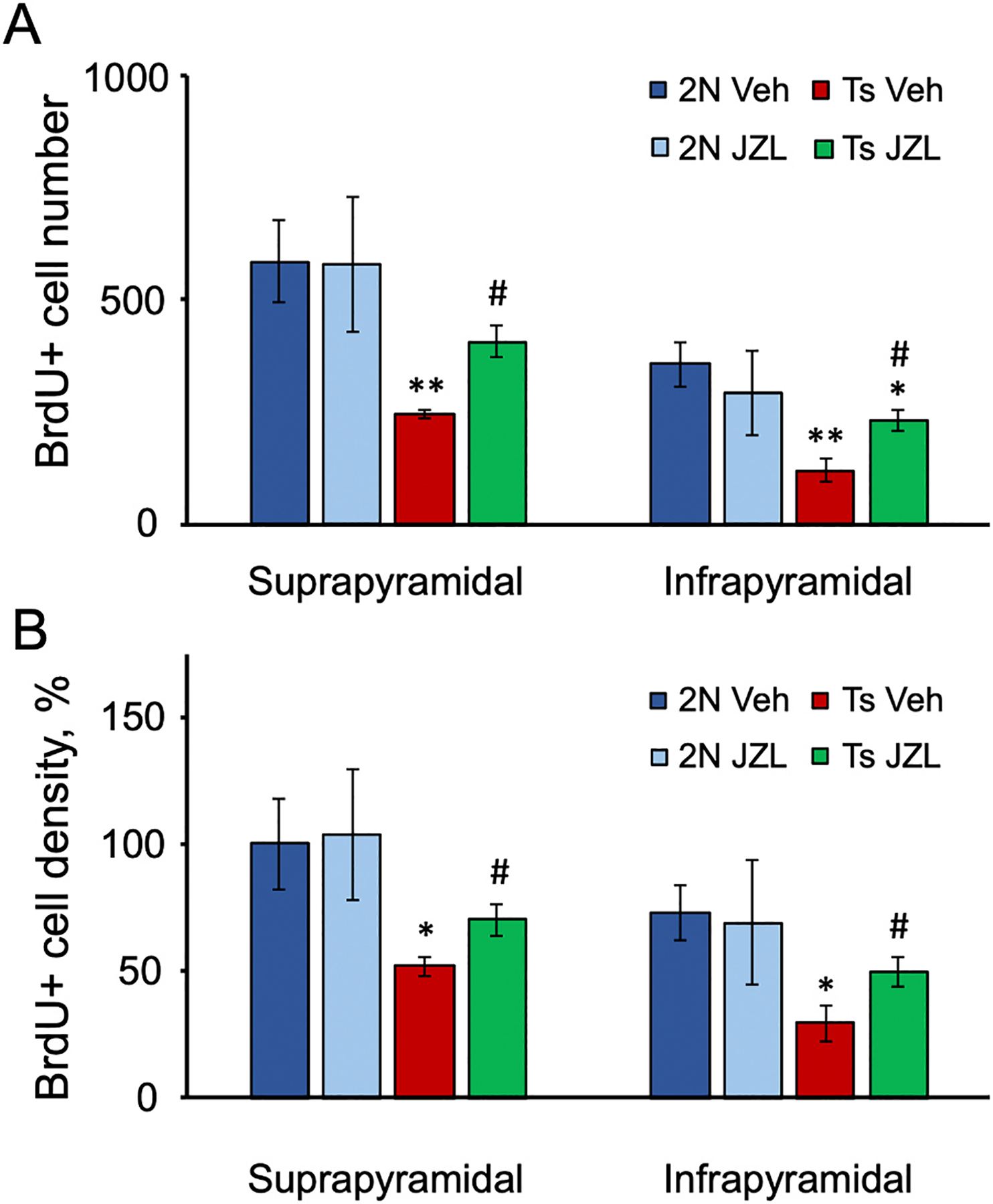
Differences in adult neurogenesis in supra- and infrapyramidal DG blades. A, B Number (A) and density (B) of BrdU-positive cells in the suprapyramidal and infrapyramidal DG blade. * p < 0.05, compared with 2 N Veh; ** p < 0.03, compared with 2 N Veh; # p < 0.05, compared with Ts Veh. **C, D** Septo-temporal changes in BrdU cell density in the suprapyramidal (C) and infrapyramidal (D) DG blade. * p < 0.05, compared with 2 N Veh; # p < 0.05, compared with Ts Veh.

DG consists of suprapyramidal and infrapyramidal blades, which differ from each other in several morphological, physiological, and other characteristics (Gallitano et al., 2016; Schmidt et al., 2012). To assess possible differences in the effects of JZL184 treatment, the total number and density of BrdU-positive cells was calculated separately for the suprapyramidal and infrapyramidal blade. Both the total number and density (Fig. 3, A and B respectively) were greater in the suprapyramidal *vs*. infrapyramidal blades, but the overall pattern of the genotype- and treatment-dependent changes seems to be similar in these blades. Indeed, the total number of BrdU-positive cells was lower in Ts Veh *vs*. 2 N Veh group and partially restored in the Ts JZL group in both blades (Fig. 3 A). Similar pattern of changes was observed for the BrdU cell density (Fig. 3 B).

To assess changes in supra- and infrapyramidal DG blades along the septo-temporal axis, the data were computed separately for the septal, intermediate, and temporal DG segments (Table 2). Again, the overall pattern of changes was similar for the suprapyramidal and infrapyramidal blade. In both blades, the BrdU cell density was reduced in Ts Veh *vs*. 2 N Veh group in all DG segments. JZL184 treatment restored the BrdU cell density in the septal DG segment of Ts65Dn mice but had little to no effect in the temporal DG segment (Table 2). Thus, adult neurogenesis was impaired in middle-aged Ts65Dn mice and restored by chronic JZL184 treatment mainly in the septal DG, in both the suprapyramidal and infrapyramidal DG blades.

## 4. Discussion

The primary aim of this study was to investigate the potential of endocannabinoid-related treatments, specifically chronic inhibition of MAGL with the selective inhibitor JZL184, in ameliorating deficits in adult hippocampal neurogenesis in the Ts65Dn mouse model of DS. We found that chronic JZL184 treatment partially restored adult hippocampal neurogenesis in Ts65Dn mice in a region-specific manner: neurogenesis was normalized in the septal DG, while remaining largely unchanged in the temporal region. This improvement was observed only in Ts65Dn mice, in which hippocampal plasticity is significantly compromised and not in normosomic (2 N) controls, suggesting that the efficacy of JZL184 depends on the underlying trisomic pathology. Overall, these findings highlight MAGL inhibition as a promising, targeted strategy for addressing hippocampal dysfunction in DS models and reveal a regionally distinct response to endocannabinoid-based therapies.

Cognitive and emotional disturbances in DS and its models are linked to widespread deficits in adult hippocampal neurogenesis. Cognitive functions depend largely on the septal (dorsal) hippocampus, while emotional processes involve the temporal (ventral) region (Anacker et al., 2018; Bienkowski et al., 2018; Fanselow and Dong, 2010; Kheirbek and Hen, 2011; Moser and Moser, 1998). In Ts65Dn mice, we observed severe impairments in neurogenesis in both regions, consistent with prior reports of reductions across the septo-temporal axis in DS models (Contestabile et al., 2013; Rueda et al., 2010). These findings highlight the broad vulnerability of neurogenesis in DS and its potential role in the full range of cognitive and emotional deficits.

The endocannabinoid system (ECS) is a promising therapeutic target for neurological, neurodevelopmental, and neurodegenerative disorders (Camma et al., 2025; Dallabrida et al., 2024; Hakami and Alshehri, 2025; Pietropaolo et al., 2020; Rezende et al., 2023). This growing interest is driven by ECS’s crucial role in brain development, synaptic plasticity, adult neurogenesis, and other processes underlying cognitive and emotional function (Prenderville et al., 2015; Xiao et al., 2023). The ECS consists of several lipid-based signaling molecules (endocannabinoids), the enzymes responsible for their biosynthesis and degradation, and the cannabinoid receptors CB1 and CB2. Therapeutic strategies include direct modulation of CB1 and CB2 receptors with exogenous agonists or antagonists, or indirect modulation by altering the activity of the enzymes that synthesize or degrade endocannabinoids (Bernal-Chico et al., 2023; League et al., 2024). The latter approach has obvious advantages, as it can increase or decrease endocannabinoid levels specifically at their sites of synthesis and release, i.e. near physiologically relevant targets, thereby reducing potential off-target effects.

The ECS is markedly dysregulated in both people with DS and mouse models. In postmortem human fetal brains, CB1 receptor (CB1R) expression is delayed by more than one month and shows abnormal developmental trajectories across multiple brain regions, including the hippocampus (Patthy et al., 2023). Later in development, CB1 levels exceed those in age-matched controls (Patthy et al., 2023). In young Ts65Dn mice, CB1R alterations are region- and cell type-specific: protein levels are elevated in the dorsal hippocampus (Di Franco et al., 2022; Navarro-Romero et al., 2019) but unchanged or slightly reduced in the ventral hippocampus (Di Franco et al., 2022). Notably, *Cnr1* mRNA is increased in dendritic layers yet reduced in the somata of CA1, CA3, and DG neurons, suggesting a post-translational compensatory regulation. These abnormalities may be amplified by overexpression of other chromosome 21 genes that modulate CB1 signaling. GIRK2 (*KCNJ6*), a key postsynaptic CB1 effector, can alter the magnitude and kinetics of CB1-mediated currents. ITSN1 and SYNJ1, which regulate clathrin-mediated endocytosis and vesicle recycling, may disrupt CB1R trafficking and surface availability. The kinase DYRK1A and the calcineurin regulator RCAN1 can influence phosphorylation–dephosphorylation cycles that control CB1R desensitization and downstream signaling. Together, these converging alterations likely exacerbate CB1R dysfunction and contribute to impaired endocannabinoid signaling in DS, suggesting that modulating endocannabinoid signaling efficiency could provide therapeutic benefits.

The regional specificity showing restoration in the septal (cognition-related) but not temporal (emotion-related) DG is highly consistent with our previous behavioral findings in Ts65Dn mice. Specifically, chronic JZL184 treatment (8 mg/kg) improved long-term recognition memory and hippocampal synaptic plasticity but demonstrated no significant effect on anxiety-linked thigmotactic behavior (Lysenko et al., 2014). These converging results support the idea that MAGL inhibition preferentially targets septal DG-dependent cognitive functions in Down syndrome, while its impact emotional behaviors associated with the temporal DG remains to be clarified in future work using dedicated anxiety and mood-related tests. Interestingly, in young Ts65Dn mice, CB1R blockade enhanced adult neurogenesis, synaptic plasticity, and memory (Navarro-Romero et al., 2019), seemingly at odds with our observation that elevating 2-AG levels *via* MAGL inhibition benefits older mice. This discrepancy likely reflects differences in age-dependent neurogenesis and ECS function, treatment strategy (enhancing compensatory 2-AG *vs*. blocking CB1R), and potential CB2R involvement in our study. Elevated 2-AG may activate CB2Rs, which have anti-inflammatory effects (Turcotte et al., 2016), potentially counteracting the neuroinflammation known to impair neurogenesis in DS (Amanollahi et al., 2023). In addition, differences in treatment duration (7 days in Navarro-Romero et al. vs. 20 days in the present study) could differentially influence CB1R signaling, as prolonged exposure to elevated 2-AG might promote CB1R desensitization and down-regulation. These findings highlight the complexity of ECS modulation in DS and suggest that therapeutic efficacy depends on age, disease stage, and the specific signaling pathways engaged.

The broader implications of our findings extend to DS - related conditions, such as Alzheimer’s disease (AD), which frequently co-occurs in DS individuals (Rafii et al., 2019). Impaired neurogenesis is a shared feature of both DS and AD, and strategies to enhance hippocampal neurogenesis have shown promise in improving cognition in AD models (Dard et al., 2019; Mu and Gage, 2011). Given that Ts65Dn mice also model aspects of AD/DS pathology (Belichenko et al., 2016; Chen et al., 2021; Kleschevnikov, A., 2022; Mojabi et al., 2016), our results suggest that MAGL inhibition could have dual relevance for cognitive deficits in DS and AD/DS. However, the lack of effect on temporal DG neurogenesis indicates that additional interventions may be needed to address emotional dysregulation, a significant clinical challenge in both conditions (Dekker et al., 2015).

An important limitation of our study is that the observed increase in dentate gyrus GCL volume in JZL184-treated Ts65Dn mice is unlikely to be fully accounted for by the modest rise in BrdU-labeled cells alone. The total number of additional BrdU+ cells represents only a very small fraction of the overall granule cell population, making it improbable that newborn neurons, by themselves, could produce the volumetric change. This observation suggests that additional structural mechanisms, potentially driven by enhanced endocannabinoid signaling, may contribute to the normalization of GCL volume. For example, MAGL inhibition and the associated increase in 2-AG could influence the morphology of pre-existing granule neurons (e.g., somatic size, dendritic arborization, or spine density) and/or modify glial and extracellular matrix components that affect tissue volume. Thus, available experimental evidence indicates that CB1 receptor activation, including under conditions of MAGL inhibition and elevated 2-AG, can promote more elaborated dendritic trees and increased axonal length and branching in hippocampal neurons, with no evidence for large changes in granule cell soma size (Compagnucci et al., 2013; Tapia et al., 2017). Such effects could potentially translate *in vivo* into an enlargement of GCL volume. Future studies employing high-resolution morphometric analyses and cell-type-specific markers will be needed to determine how JZL184 alters granule neuron and glial architecture in the dentate gyrus and how these changes relate to the restored GCL volume.

To precisely define the stage of adult neurogenesis selectively enhanced by chronic MAGL inhibition, future studies must employ cell-fate markers beyond BrdU labeling. This will include: (i) Ki-67 immunohistochemistry at the time of sacrifice to quantify the proliferating pool and determine whether the proliferative effect is sustained after the end of BrdU administration; (ii) double-labeling of BrdU with Nestin to identify proliferating neural stem/progenitor cells; and (iii) double-labeling of BrdU with Doublecortin (DCX) and/or PSA-NCAM to quantify immature granule neurons generated during the treatment period. These approaches will clarify whether JZL184 primarily enhances proliferation, early survival, or neuronal differentiation. Furthermore, the pronounced septo-temporal bias observed in our BrdU data requires further regional molecular analysis. Future work will assess the septal versus temporal distribution of key endocannabinoid system components, including MAGL, CB1R, and CB2R, within the DG to uncover mechanisms underlying the regional specificity of the response to MAGL inhibition. Finally, to test the hypothesis suggested by the GCL volumetric changes, detailed morphometric analyses of mature granule neurons (somatic size and dendritic arborization) will be required to determine whether JZL184 alters DG cytoarchitecture in addition to increasing BrdU-labeled cell number.

An additional, potentially important level of regulation involves astrocytes. Astrocytes in the dentate gyrus express CB1, CB2, and TRPV1 receptors and are responsive to endocannabinoids, and astrocyte-derived signals have been shown to modulate the activation of quiescent NSCs and their progression to type 2a/2b/3 progenitors, in part *via* ephrin-B signaling (Ashton et al., 2012). The strong septal bias of the proliferative response raises the possibility that regional differences in astrocyte abundance or ephrin-B2 expression along the septo-temporal axis contribute to the selective restoration of neurogenesis in the septal DG. Future experiments will therefore examine septo-temporal gradients in astrocyte density and ephrin-B2 expression, and their relationship to NSC activation under MAGL inhibition.

In conclusion, our study demonstrates that chronic suppression of MAGL with JZL184 partially restores adult neurogenesis in the septal DG of middle-aged Ts65Dn mice, providing a potential mechanism for the previously observed improvements in synaptic plasticity and cognition. These findings highlight the therapeutic potential of targeting the endocannabinoid system to mitigate cognitive impairment in DS, and possibly AD/DS, while underscoring the need for further research into age-specific effects, regional specificity, and the contributions of CB1R versus CB2R signaling. By selectively enhancing neurogenesis in a cognition-related hippocampal subregion, MAGL inhibitors like JZL184 could offer a precision approach to treating neurodevelopmental disorders characterized by hippocampal dysfunction.

## Figures and Tables

**Table 1:** Gross-morphological parameters of the DG granule cell layer (GCL).

	2 N Veh	2 N JZL	Ts Veh	Ts JZL
GCL Volume (mm^3^)	0.337 ± 0.010	0.394 ± 0.016	0.287 ± 0.01*	0.379 ± 0.019^#^
GCL Volume (%)	100.0 ± 2.7	104.4 ± 4.3	76.3 ± 2.9*	100.5 ± 5.2^#^
GCL Width (μm)	68.2 ± 1.9	72.0 ± 5.3	65.7 ± 2.5	66.5 ± 2.2
GCL Width (%)	100. 0 ± 2.8	105.5 ± 7.8	96.3 ± 3.7	97.5 ± 3.2

Values are expressed in absolute values and as % of 2 N Veh (set to 100 %). GCL volume was significantly reduced in Ts65Dn Veh vs. 2 N Veh and normalized by JZL184 in Ts65Dn. JZL184 had no effect in 2 N mice. GCL width showed no significant differences across groups. Data are mean ± SEM.

* p < 0.03 vs. 2 N Veh

# p < 0.03 vs. Ts Veh.

**Table 2:** BrdU+ cell density across DG subregions.

	2 N Veh	2 N JZL	Ts Veh	Ts JZL
Suprapyramidal DG blade				
Septal	100.0 ± 20.8	98.4 ± 28.9	43.1 ± 8.1*	91.2 ± 12.9^#^
Middle	100.0 ± 13.7	117.9 ± 23.6	57.5 ± 5.7*	68.6 ± 7.4
Temporal	100.0 ± 19.9	99.6 ± 33.8	61.3 ± 7.2*	59.4 ± 1.6^&^
Infrapyramidal DG blade				
Septal	100.0 ± 21.4	87.0 ± 28.5	45.1 ± 10.4*	72.3 ± 18.6
Middle	100.0 ± 16.0	107.4 ± 21.0	48.3 ± 23.7	82.6 ± 15.1
Temporal	100.0 ± 21.9	87.3 ± 30.8	31.3 ± 6.7*	52.3 ± 6.0^#^

Values are % of 2 N Veh (set to 100 %). Both suprapyramidal and infrapyramidal blades showed reduced BrdU+ density in Ts65Dn Veh mice across all segments. JZL184 restored density primarily in the septal DG, with little to no effect in the temporal DG. Data are mean ± SEM.

* p < 0.03 vs. 2 N Veh

# p < 0.03 vs. Ts Veh.

## Abbreviations

ACSF: Artificial cerebrospinal fluid
AD: Alzheimer’s disease
BrdU: bromodeoxyuridine
DG: dentate gyrus
DS: Down syndrome
ECS: endocannabinoid system
GCL: granule cell layer
MAGL: monoacylglycerol lipase
NHS: normal horse serum
OCT: optimal cutting temperature medium
PFA-PBS: phosphate-buffered saline
SGZ: subgranular zone
2-AG: 2-arachidonoylglycerol
